# Ebola Virus Disease: Uniquely Challenging Among the Viral Hemorrhagic Fevers

**DOI:** 10.1093/ofid/ofaf464

**Published:** 2025-08-02

**Authors:** M Jeremiah Matson, Daniel S Chertow, Vincent J Munster

**Affiliations:** Division of Infectious Diseases, Department of Internal Medicine, University of Utah, Salt Lake City, Utah, USA; Laboratory of Virology, Rocky Mountain Laboratories, National Institute of Allergy and Infectious Diseases, National Institutes of Health, Hamilton, Montana, USA; Critical Care Medicine Department, National Institutes of Health Clinical Center, Bethesda, Maryland, USA; Laboratory of Virology, Rocky Mountain Laboratories, National Institute of Allergy and Infectious Diseases, National Institutes of Health, Hamilton, Montana, USA

**Keywords:** Ebola, Ebola virus disease, Filoviridae, filovirus, viral hemorrhagic fever

## Abstract

Viral hemorrhagic fever is a severe illness characterized by constitutional signs and symptoms accompanied by coagulopathy, shock, and multiorgan failure caused by dozens of enveloped RNA viruses spanning 6 viral families: Flaviviridae, Arenaviridae, Hantaviridae, Nairoviridae, Phenuiviridae, and Filoviridae. Ebola virus (EBOV), the etiologic agent of Ebola virus disease (EVD), is among the deadliest and accounts for the majority of known human infections and deaths within the family Filoviridae. EBOV was responsible for the 2013–2016 West Africa epidemic and the 2018–2020 Democratic Republic of the Congo epidemic, both of which were declared public health emergencies of international concern by the World Health Organization. The ecology of Ebola virus is poorly characterized, with its animal reservoir and drivers of zoonotic spillover unknown. Once spillover has occurred, EBOV's human-to-human transmission makes containment challenging and poses significant nosocomial risk. Vaccines and targeted therapeutics have been developed, tested, and approved by regulatory agencies over the past decade, but some uncertainty remains regarding efficacy. Infrastructure is often insufficient to effectively provide efficient public health responses and advanced supportive clinical care in EVD outbreak areas. Further research of EBOV in closer proximity to areas most affected by EVD is needed, but the containment facilities required for such work require nontrivial investment and personnel. These factors combine to make EBOV a uniquely challenging virus and cannot be easily overcome. The escalation of impacts from EVD over the past decade serves as a warning, however, that approaching these challenges should not wait until the next major outbreak.

Viral hemorrhagic fevers (VHFs) present clinically with constitutional signs and symptoms accompanied by coagulopathy (with or without overt hemorrhage), shock, and multiorgan failure [1]. Depending on the etiologic agent, a broad spectrum of additional findings may be present, including pulmonary, gastrointestinal, ocular, and neurologic manifestations. Many dozens of enveloped RNA viruses are currently known to cause VHF, with more being discovered regularly [2]. At present, these viruses span 6 families: Arenaviridae, Filoviridae, Flaviviridae, Hantaviridae, Nairoviridae, and Phenuiviridae. Infection with many of these viruses typically results in only mild illness; it may even be asymptomatic, and it rarely progresses to fulminant hemorrhagic fever. Infection with others, such as Ebola virus (EBOV), regularly leads to the most severe and life-threatening phenotype of the syndrome, with case fatality rates often exceeding 50%. Some of the viruses that cause VHF have single-digit reported case numbers, such as Sabiá virus and Taï Forest virus, while others are perennial scourges that have cumulatively been responsible for countless millions of infections, including Lassa virus (LASV), yellow fever virus (YFV), and dengue virus (DENV). Among the viruses in the family Filoviridae, EBOV is the most familiar as it was the etiologic agent of the West Africa epidemic from 2013 to 2016. This was the largest Ebola virus disease (EVD) outbreak by far, with approximately 30 000 cases reported, and it was declared a public health emergency of international concern by the World Health Organization during that time. The second-largest outbreak occurred shortly afterward from 2018 to 2020 in the Democratic Republic of the Congo (DRC) with nearly 3500 cases reported, once again meriting declaration as a public health emergency of international concern. Considering these events, increased investment has been made over the past decade to further our understanding of and preparedness for the threats that EBOV poses. Yet, it remains an enigmatic virus in many respects and continues to pose significant risks to human health. This review places EBOV within the greater context of the other viruses that cause VHF and explores various factors that combine to set it apart as uniquely challenging.

## VIRUSES THAT CAUSE VHF

**Figure 1.**
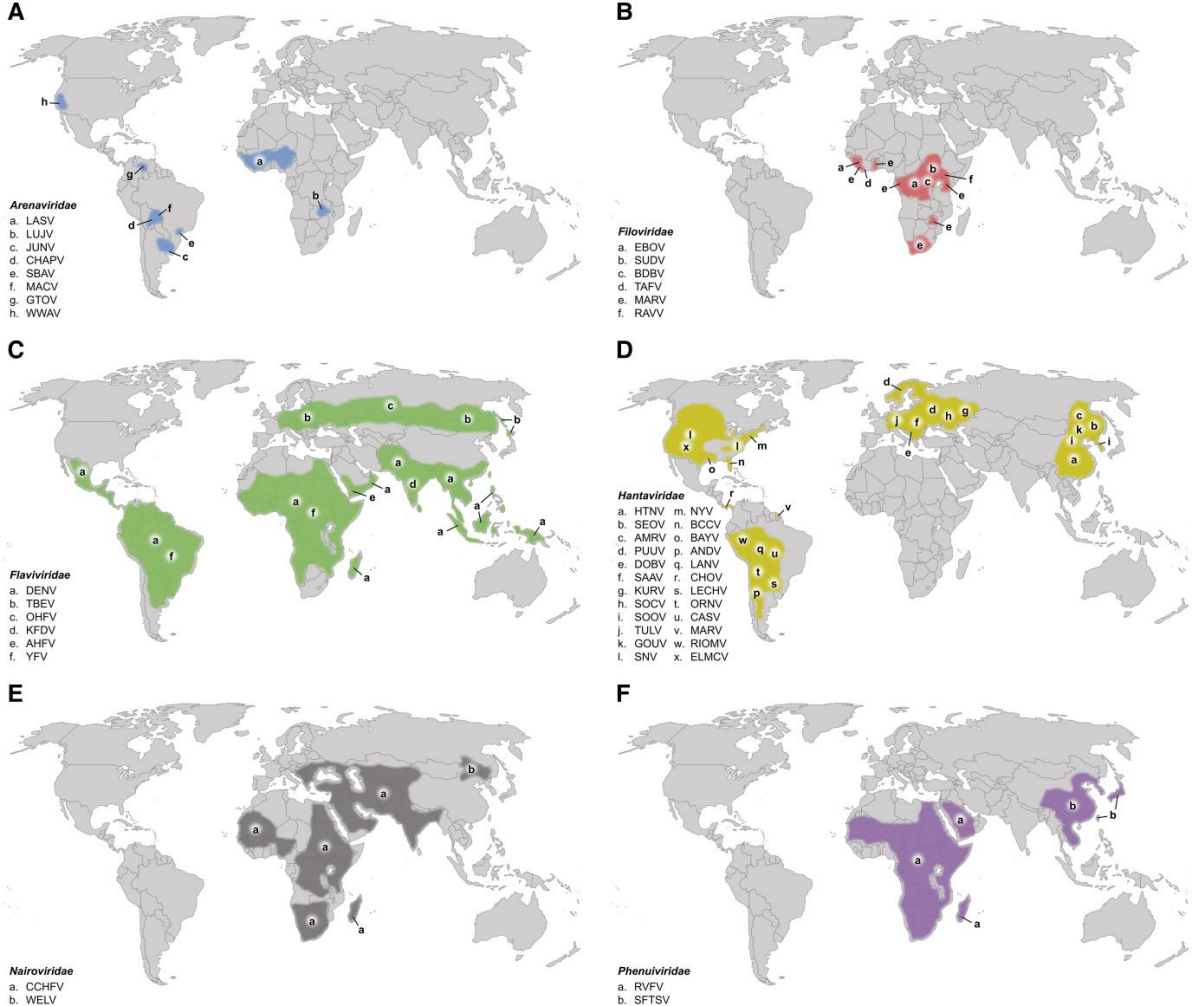
Global map of the known spillover ranges of the hemorrhagic fever viruses by family: *A*, Arenaviridae; *B*, Filoviridae; *C*, Flaviviridae; *D*, Hantaviridae; *E*, Nairoviridae; *F*, Phenuiviridae. The highlighted areas for each virus family generally approximate the range for which specific viruses, designated by the letters corresponding to the key, have caused human cases of viral hemorrhagic fever. Some letters are present more than once on the maps if the known spillover range of a given virus is broad or discontinuous. For the family Hantaviridae, viruses not known to have caused human disease and those without formal International Committee on Taxonomy of Viruses classification at the time of publication have generally been excluded [103]. SEOV has been identified as the causative agent of hemorrhagic fever with renal syndrome in numerous countries around the world, including the United States; however, most cases outside of East Asia have been associated with pet rats, and those locations are not indicated on the map [52]. Virus abbreviations are as follows. Arenaviridae: LASV, Lassa virus; LUJV, Lujo virus; JUNV, Junín virus; CHAPV, Chapare virus; SBAV, Sabiá virus; MACV, Machupo virus; GTOV, Guanarito virus; WWAV, Whitewater Arroyo virus. Filoviridae: EBOV, Ebola virus; SUDV, Sudan virus; BDBV, Bundibugyo virus; TAFV, Taï Forest virus; MARV, Marburg virus; RAVV, Ravn virus. Flaviviridae: DENV, dengue virus; TBEV, tick-borne encephalitis virus; OHFV, Omsk hemorrhagic fever virus; KFDV, Kyasanur Forest disease virus; AHFV, Alkhumra hemorrhagic fever virus; YFV, yellow fever virus. Hantaviridae: HTNV, Hantaan virus; SEOV, Seoul virus; AMRV, Amur virus; PUUV, Puumala virus; DOBV, Dobrava virus; SAAV, Saaremaa virus; KURV, Kurkino virus; SOCV, Sochi virus; SOOV, Soochong virus; TULV, Tula virus; GOUV, Gōu virus; SNV, Sin Nombre virus; NYV, New York virus; BCCV, Black Creek Canal virus; BAYV, Bayou virus; ANDV, Andes virus; LANV, Laguna Negra virus; CHOV, Choclo virus; LECHV, Lechiguanas virus; ORNV, Orán virus; CASV, Castelo dos Sonhos virus; MARV, Maripa virus; RIOMV, Rio Mamore virus; ELMCV, El Moro Canyon virus. Nairoviridae: CCHFV, Crimean-Congo hemorrhagic fever virus; WELV, wetland virus. Phenuiviridae: RVFV, Rift Valley fever virus; SFTSV, severe fever with thrombocytopenia syndrome virus.

**Figure 2.**
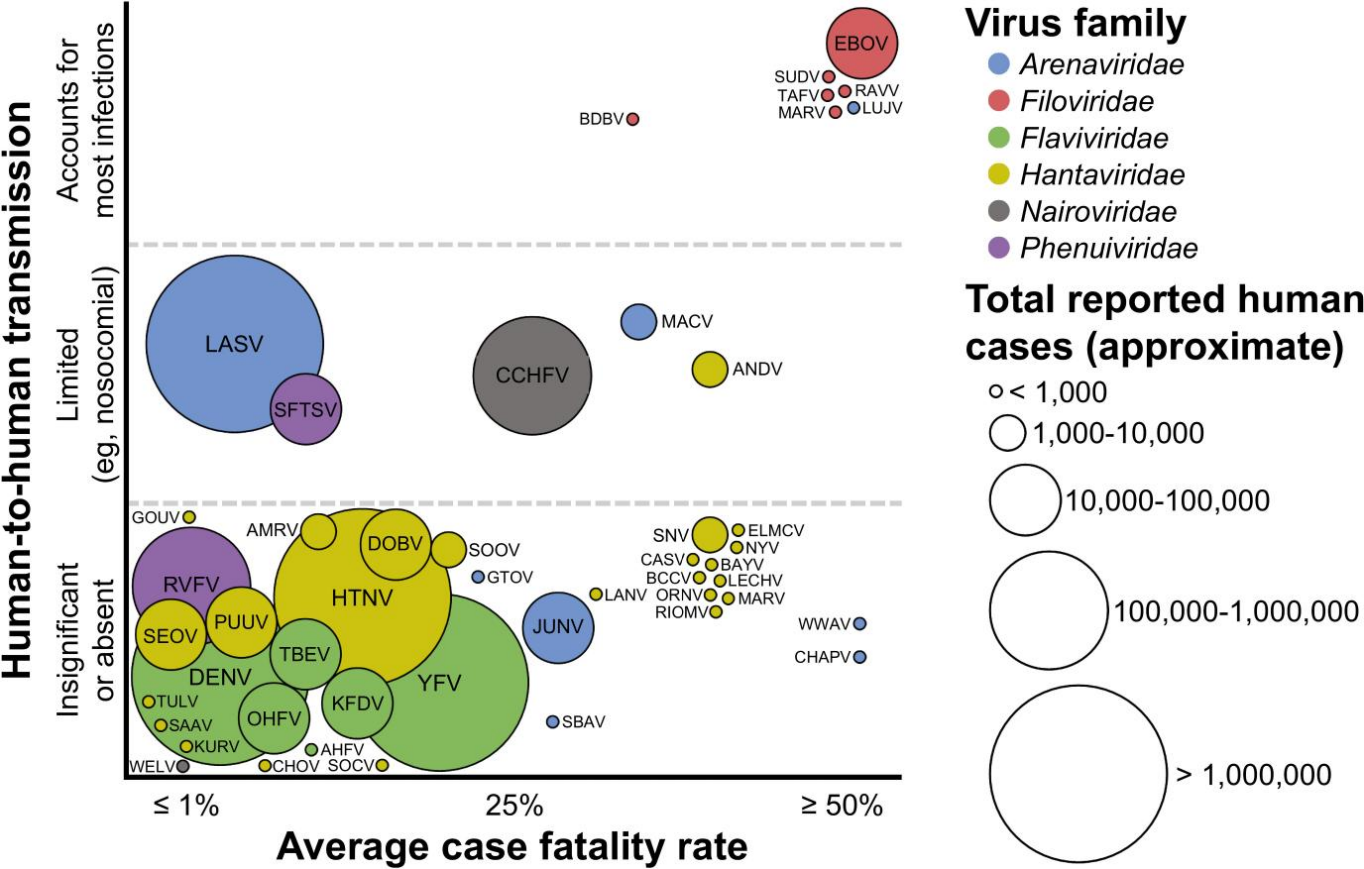
Average case fatality rates, approximate cumulative known cases, and degree of direct human-to-human transmission for the hemorrhagic fever viruses by family. The vertical divisions along the y-axis are discrete categories (ie, a position higher or lower within a given transmission category does not have any significance). The x-axis is a spectrum from ≤1% to ≥50%. The case fatality rates as shown indicate the overall estimated case fatality rates for all infections with a given virus, not exclusively those infections that progress to hemorrhagic fever. Approximate cumulative known cases are indicated by circle size. For viruses that have caused <1000 cumulative cases of viral hemorrhagic fever, many are responsible for only single-digit cases; as such, case fatality rates may not be reliable given a small sample size. Total numbers of cases were estimated from available literature; see the references listed in Table 1. For the family Hantaviridae, viruses not known to have caused human disease and those without formal International Committee on Taxonomy of Viruses classification at the time of publication have generally been excluded [103]. Virus abbreviations are as follows. Arenaviridae: CHAPV, Chapare virus; GTOV, Guanarito virus; JUNV, Junín virus; LASV, Lassa virus; LUJV, Lujo virus; MACV, Machupo virus; SBAV, Sabiá virus; WWAV, Whitewater Arroyo virus. Filoviridae: BDBV, Bundibugyo virus; EBOV, Ebola virus; MARV, Marburg virus; RAVV, Ravn virus; SUDV, Sudan virus; TAFV, Taï Forest virus. Flaviviridae: AHFV, Alkhumra hemorrhagic fever virus; DENV, dengue virus; KFDV, Kyasanur Forest disease virus; OHFV, Omsk hemorrhagic fever virus; TBEV, tick-borne encephalitis virus; YFV, yellow fever virus. Hantaviridae: AMRV, Amur virus; ANDV, Andes virus; BAYV, Bayou virus; BCCV, Black Creek Canal virus; CASV, Castelo dos Sonhos virus; CHOV, Choclo virus; DOBV, Dobrava virus; ELMCV, El Moro Canyon virus; GOUV, Gōu virus; HTNV, Hantaan virus; KURV, Kurkino virus; LANV, Laguna Negra virus; LECHV, Lechiguanas virus; MARV, Maripa virus; NYV, New York virus; ORNV, Orán virus; PUUV, Puumala virus; RIOMV, Rio Mamore virus; SAAV, Saaremaa virus; SEOV, Seoul virus; SNV, Sin Nombre virus; SOCV, Sochi virus; SOOV, Soochong virus; TULV, Tula virus. Nairoviridae: CCHFV, Crimean-Congo hemorrhagic fever virus; WELV, wetland virus. Phenuiviridae: RVFV, Rift Valley fever virus; SFTSV, severe fever with thrombocytopenia syndrome virus.

The family Arenaviridae includes LASV, which is endemic in West Africa (Table 1, Figure 1*A*). LASV may cause millions of infections annually, although only 20% of these are symptomatic and the overall case fatality rate of LASV infection with fulminant VHF is approximately 1% (Figure 2) [104]. Other viruses within the family Arenaviridae that can cause VHF are found in Africa and North and South America and are generally far less common but have higher case-fatality rates. All viruses within the family have rodents as their natural reservoirs and generally transmit to humans by contact with an infected animal, either from handling or from exposure to excrement or other bodily fluids. LASV can transmit directly from human to human, although it does so poorly [105]. Machupo virus is also capable of direct human-to-human transmission, as is Lujo virus based on the single small outbreak that has been recorded [24, 26].

**Table 1.** Viruses Known to Cause Viral Hemorrhagic Fever

Family: Species	Member Viruses	Known Reservoirs	Spillover Mechanism	Nonhuman Impacts
Flaviviridae				
*Orthoflavivirus denguei*	DENV [3–7]	Hard-body ticks (*Dermacentor* spp, OHFV; *Hemaphysalis spinigera*, KFDV; *Hyalomma dromedarii*, AHFV; *Ixodes* spp, TBEV, OHFV), soft-body *Ornithodoros savigyni* ticks (AHFV), small rodents (TBEV, OHFV), and nonhuman primates (DENV, YFV)	Direct contact with infected animals or animal products (AHFV, KFDV, OHFV, TBEV), tick bites (AHFV, KFDV, OHFV, TBEV), and mosquito bites (DENV, YFV)	High-mortality epizootics among nonhuman primates (KFDV)
*Orthoflavivirus kyasanurense*	AHFV [8–10], KFDV [11, 12]			
*Orthoflavivirus omskense*	OHFV [13]			
*Orthoflavivirus encephalitidis*	TBEV [14, 15]			
*Orthoflavivirus flavi*	YFV [16, 17]			
Arenaviridae				
*Mammarenavirus chapareense*	CHAPV [18, 19]	Small rodents (various species)	Direct or indirect (eg, secretions, excretions) contact with small rodents	None reported
*Mammarenavirus guanaritoense*	GTOV [19, 20]			
*Mammarenavirus juninense*	JUNV [19–21]			
*Mammarenavirus lassaense*	LASV [19, 20, 22, 23]			
*Mammarenavirus lujoense*	LUJV [19, 24, 25]			
*Mammarenavirus machupoense*	MACV [19, 20, 26, 27]			
*Mammarenavirus brazilense*	SBAV [19, 20, 28]			
*Mammarenavirus whitewaterense*	WWAV [29, 30]			
Hantaviridae^a^				
Old World				
*Orthohantavirus dobravaense*	DOBV [31–33], KURV [34, 35], SAAV [33, 36, 37], SOCV [38, 39]	Small rodents (various species)	Direct or indirect (eg, secretions, excretions) contact with small rodents; limited direct human-to-human transmission for ANDV	None reported
*Orthohantavirus hantanense*	AMRV [40, 41], HTNV [38, 42–44], SOOV [45, 46]			
*Orthohantavirus puumalaense*	PUUV [47, 48]			
*Orthohantavirus seoulense*	GOUV [38, 49]SEOV [50–52]			
*Orthohantavirus tulaense*	TULV [53, 54]			
New World				
*Orthohantavirus sinnombreense*	SNV [55, 56], NYV [57]			
*Orthohantavirus bayoui*	BAYV [58]			
*Orthohantavirus nigrorivense*	BCCV [56, 59, 60]			
*Orthohantavirus andesense*	ANDV [61, 62], CASV [63], LECHV [64–66], ORNV [67]			
*Orthohantavirus negraense*	LANV [68–70], RIOMV [71], MARV (Maripa virus) [72]			
*Orthohantavirus chocloense*	CHOV [73]			
*Orthohantavirus moroense*	ELMCV [74]			
Nairoviridae				
*Orthonairovirus* sp (newly discovered)	WELV [2]	Hard-body ticks of the family Ixodidae	Tick bites and/or contact with infected livestock	None reported
*Orthonairovirus haemorrhagiae*	CCHFV [75–78]			
Phenuiviridae				
*Phlebovirus riftense*	RVFV [9, 79, 80]	Mosquitoes (primarily of genus *Aedes*) for RVFV; hard-body ticks (primarily of genus *Haemaphysalis*) for SFTSV	Mosquito bites, fly bites, and/or contact with infected animals or animal products for RVFV; tick bites (primarily) or, rarely, direct human-to-human for SFTSV	High-mortality epizootics among livestock
*Bandavirus dabieense*	SFTSV [81–84]			
Filoviridae				
*Orthomargburgvirus marburgense*	MARV (Marburg virus) [85–91], RAVV [85, 92–94]	Unknown for ebolaviruses; frugivorous and/or insectivorous bats suspected; *Rousettus aegyptiacus* frugivorous bats for MARV	Unclear; direct contact with intermediate hosts or reservoir hosts suspected	High-mortality epizootics among nonhuman primates and possibly other mammals
*Orthoebolavirus bundibugyoense*	BDBV [95, 96]			
*Orthoebolavirus sudanense*	SUDV [93, 97, 98]			
*Orthoebolavirus taiense*	TAFV [99]			
*Orthoebolavirus zairense*	EBOV [100–102]			

Abbreviations: AHFV, Alkhumra hemorrhagic fever virus; AMRV, Amur virus; ANDV, Andes virus; BAYV, Bayou virus; BCCV, Black Creek Canal virus; BDBV, Bundibugyo virus; CASV, Castelo dos Sonhos virus; CCHFV, Crimean-Congo hemorrhagic fever virus; CHAPV, Chapare virus; CHOV, Choclo virus; DENV, dengue virus; DOBV, Dobrava virus; EBOV, Ebola virus; ELMCV, El Moro Canyon virus; GOUV, Gōu virus; GTOV, Guanarito virus; HTNV, Hantaan virus; JUNV, Junín virus; KFDV, Kyasanur Forest disease virus; KURV, Kurkino virus; LANV, Laguna Negra virus; LASV, Lassa virus; LECHV, Lechiguanas virus; LUJV, Lujo virus; MACV, Machupo virus; MARV, Marburg virus or Maripa virus; NYV, New York virus; OHFV, Omsk hemorrhagic fever virus; ORNV, Orán virus; PUUV, Puumala virus; RAVV, Ravn virus; RIOMV, Rio Mamore virus; RVFV, Rift Valley fever virus; SAAV, Saaremaa virus; SBAV, Sabiá virus; SEOV, Seoul virus; SFTSV, severe fever with thrombocytopenia syndrome virus; SNV, Sin Nombre virus; SOCV, Sochi virus; SOOV, Soochong virus; SUDV, Sudan virus; TAFV, Taï Forest virus; TBEV, tick-borne encephalitis virus; TULV, Tula virus; WELV, wetland virus; WWAV, Whitewater Arroyo virus; YFV, yellow fever virus.

^a^For the family Hantaviridae, viruses not known to have caused human disease and those without formal International Committee on Taxonomy of Viruses classification at the time of publication have generally been excluded [103].

The family Filoviridae includes 6 viruses known to cause VHF, including EBOV, all within Africa (Table 1, Figure 1*B*). Three of the viruses, in addition to EBOV, are collectively known as “ebolaviruses”: Sudan virus, Bundibugyo virus, and Taï *Forest* virus. The remaining 2 are different member viruses of the same species, *Orthomarburgvirus margburgense*: Marburg virus (MARV) and Ravn virus (RAVV). EBOV and MARV/RAVV occur across a broader geographic area (based on spillovers) than the other viruses. All spread essentially exclusively via direct human-to-human transmission following sporadic zoonotic spillover events from unidentified reservoir species—except for MARV/RAVV, for which the reservoir is known to be *Rousettus aegyptiacus* bats (Figure 2) [85, 106]. EBOV has caused nearly 40 000 human infections since it was first recognized in 1976, whereas all other viruses within the family Filoviridae have collectively accounted for approximately 1500 human infections [107]. The reasons for this vast discrepancy are unknown. Additionally, many of the viruses in the family Filoviridae have been associated with devastating mortality events among nonhuman primates and other wildlife [108].

The viruses of the family Flaviviridae include well-known examples such as DENV and YFV (Table 1). DENV has cumulatively caused hundreds of millions of confirmed human infections and is endemic across much of the world in tropical and subtropical regions (Figure 1*C*) [109]. Infection with DENV rarely progresses to a syndrome consistent with VHF, however, and case fatality rates are generally <1% (Figure 2). YFV is the deadliest of the viruses that cause VHF in the family Flaviviridae, with case fatality rates often exceeding 20% [110]. Most cases occur in sub-Saharan Africa and South America. All viruses of the family Flaviviridae that cause VHF are transmitted by an arthropod vector regardless of whether the transmission is between wild animals (sylvatic cycle), between humans (urban cycle), or between wild animals and humans (intermediate cycle); some may transmit occasionally via direct contact with infected animals or animal products [8, 14, 15]. No viruses within the family Flaviviridae are currently known to be capable of direct human-to-human transmission.

Viruses within the family Hantaviridae are generally divided between Old World viruses, present in Europe and Asia, and New World viruses, present in North and South America (Table 1, Figure 1*D*). Infection with Old World hantaviruses produces a syndrome termed *hemorrhagic fever with renal syndrome*. Case fatality rates generally range from 1% to 15% depending on the causative virus [111]. Infection with New World hantaviruses typically results in a more deadly syndrome termed *hantavirus pulmonary syndrome*, also known as *hantavirus cardiopulmonary syndrome*, and case fatality rates usually range from 25% to 50%. The notable exception is Choclo virus in Panama, which has much lower pathogenicity [73] (Figure 2). Hemorrhagic fever with renal syndrome and hantavirus pulmonary/cardiopulmonary syndrome are more broadly considered to be VHFs [112]. All viruses within the family Hantaviridae are transmitted to humans by small rodents, usually via exposure to their aerosolized excrement. Current evidence suggests that the Andes virus is unique within the family Hantaviridae in its ability to subsequently transmit directly from human to human following zoonotic spillover [61, 62].

The family Nairoviridae currently comprises only 2 viruses that cause VHF: Crimean-Congo hemorrhagic fever virus in Africa, the Middle East, and parts of Europe and Asia and the recently described wetland virus in northeastern China [2] (Table 1, Figure 1*E*). Both rely on ticks for transmission between wild and domestic animals and ultimately to humans, although Crimean-Congo hemorrhagic fever virus may transmit via direct contact with numerous species of subclinically infected animals or rarely directly from human to human, mostly in health care settings (Figure 2) [75].

Similarly, the family Phenuiviridae currently has 2 member viruses that cause VHF: Rift Valley fever virus, present in Africa and the Arabian Peninsula, and severe fever with thrombocytopenia syndrome virus, present in East Asia (Table 1, Figure 1*F*). Both viruses are dependent on arthropod vectors for transmission between animals and from animals to humans: Rift Valley fever virus by mosquitoes and severe fever with thrombocytopenia syndrome virus by ticks. Rift Valley fever virus is notable for its nonhuman impacts in that it causes significant morbidity and mortality among livestock, particularly sheep and goats, and can be transmitted to humans via contact with infected animals or animal products (eg, raw milk or undercooked meat) [113]. Severe fever with thrombocytopenia syndrome virus is associated with a higher case fatality rate and is known to occasionally transmit directly from human to human, with some evidence suggesting that sexual transmission may be rarely implicated [81] (Figure 2).

## CHALLENGES IN CHARACTERIZING THE ECOLOGY OF EBOV

The reservoir/intermediate hosts and vectors (where applicable) for most of the hemorrhagic fever viruses are known, with the notable exception of those within the family Filoviridae (the filoviruses; Table 1). Of the filoviruses that are known to be pathogenic to humans, only MARV and RAVV have been definitively associated with a natural reservoir host—frugivorous *R aegyptiacus* bats—by virus isolation from the bats and evidence suggesting continuous circulation of MARV within these bats in Uganda [114]. EBOV serologic surveys have been broadly undertaken among wildlife. Anti-EBOV antibodies have been reported among *Epomops franqueti*, *Hypsignathus monstrosus*, *Myonycteris torquate*, *Micropteropus pusillus*, and *R aegyptiacus* bats in Gabon [115, 116]; *Eidolon helvum*, *E franqueti*, *Epomophorus gambianus*, and *H monstrosus* bats in Ghana [117]; *Artibeus lituratus* bats in Trinidad and Tobago [118]; and *Rousettus leschenaultii* bats in Bangladesh [119]. Many of the serologically positive samples in these studies demonstrated cross-reactivity to other filoviruses (eg, Reston virus) and often had somewhat discrepant results if various techniques or assays were utilized (eg, enzyme-linked immunosorbent assay vs Western blot). Detection of EBOV RNA in bats has rarely been accomplished, with examples including positive results by polymerase chain reaction (PCR) of liver and spleen homogenate from *E franqueti*, *H monstrosus*, and *M torquate* bats in Gabon [115, 116] and by an oral swab in a single carnivorous *Miniopterus inflatus* bat in Liberia in 2019 [120], although this latter result has not been published in a peer-reviewed journal to date. Definitive identification of the natural reservoirs of EBOV may inform rational public health efforts to mitigate spillover events, and ongoing research is essential.

Evidence suggests that various mammals, including nonhuman primates, may act as amplifying intermediate hosts in the transmission of EBOV to humans. Serologic studies have identified anti-EBOV antibodies in numerous nonhuman primates, including *Gorilla gorilla*, *Pan troglodytes*, *Mandrillus* spp, *Cercopithecus neglectus*, *Papio anubis*, as well as *Cephalophus* spp (antelopes) [108, 121]. One study found that 17.6% (21/119) of blood samples from wild-born chimpanzees in Cameroon were positive, while all captive chimpanzee samples were negative. Additionally, seropositive nonhuman primate samples obtained from the well-established outbreak areas of Gabon and the Republic of the Congo dated back to 1985 and 1996, respectively—both substantially predating the first reported outbreaks of human EVD in these countries [121]. EBOV is also likely equally devastating to these populations and is suspected to have caused the death of innumerable numbers of these animals, including up to 5000 gorillas just between 2002 and 2003 [108, 121–123]. Efforts have been made to educate hunters of the importance of notifying authorities when animal carcasses are found, and point-of-care testing has been developed and deployed to facilitate rapid identification of possible EBOV infections in animals [124]. Nevertheless, finding ways to protect endangered wildlife from the threat of EBOV is an immense challenge that, at present, has few meaningful pathways forward.

## CHALLENGES POSED BY EBOV TRANSMISSION

Viruses of the family Filoviridae are generally unique among the viruses that cause VHF in their degree of direct human-to-human transmissibility, with only Lujo virus of the family Arenaviridae possibly comparable (Figure 2). Just 5 cases of VHF due to Lujo virus from a single outbreak are currently known, however, so its transmission dynamics remain poorly characterized [25]. For EBOV, transmission during an outbreak is directly human to human following an initial zoonotic spillover event, as is the case for other viruses within the family Filoviridae [106]. Although the infectious dose for EBOV is not precisely known, it may be as low as ≤10 infectious virus particles for humans [125]. Nosocomial transmission is of particular concern, with data indicating that health care workers (HCWs) were 40 times more likely to become infected with EBOV than non-HCWs in Guinea [126]. More than 850 HCWs were infected with EBOV in West Africa, resulting in >500 deaths [127]. Deaths among HCWs impede efforts to quell outbreaks by reducing the workforce and discouraging those who remain from continuing to contribute.

Recognizing the index case of EVD is hampered by the nonspecific signs and symptoms present early in infection and by inadequate access to molecular and/or point-of-care diagnostic testing. Once an EVD outbreak or epidemic is recognized, however, transmission chains in EVD are often readily identified by epidemiologic investigation, with new cases typically linked to providing care for a sick family member or participating in funeral rituals. Interestingly, though, numerous cases during EVD outbreaks have no identifiable exposures or risk factors, suggesting that in some instances humans may be infected and possibly even contagious despite being asymptomatic or paucisymptomatic [128, 129]. While most evidence in support of this is serologic, 1 study reported 7 persons from the 1996 EVD outbreak in Gabon who were positive for EBOV by PCR yet entirely asymptomatic [130]. Virus could not be isolated from the patient samples, and detection of viral RNA was possible only with a nested PCR on extract from peripheral blood mononuclear cells, suggesting a very low level of replication and viremia. The significance of these data is unclear, but they could have important ramifications for understanding EVD pathogenesis and epidemiology, particularly if asymptomatic or paucisymptomatic individuals are capable of transmitting virus under some circumstances.

A related scenario that is of concern is the transmission of EBOV during convalescence or even after full recovery from EVD. After the acute phase of disease, EBOV persists in humans in numerous tissues, including the eye, the central nervous system, the testes, and possibly female breast tissue [131–134]. Persistent infection in the testes is particularly worrisome, as male-to-female sexual transmission has been reported on numerous occasions, in 1 instance occurring more than a year after male convalescence [133, 135, 136]. During the 2021 resurgence of EVD in Guinea, more than 5 years after the end of the West Africa EVD epidemic, molecular evidence revealed suspected transmission from a convalescent patient with persistent infection. Full genome sequencing demonstrated well-supported phylogenetic clustering of the 2021 viral sequences with EBOV lineages from Guinea in 2013 to 2016. A thorough epidemiologic investigation was unrevealing, and this raised speculation that intermittent asymptomatic shedding of infectious virus by routes other than male-to-female sexual transmission could be possible [137]. Aside from a single case of male-to-female sexual transmission that has been reported for MARV, also within the family Filoviridae, and relatively weak evidence suggesting sexual transmission in 3 patients infected with Crimean-Congo hemorrhagic fever virus of the family Nairoviridae, such phenomena are not known to occur among the other viruses that cause VHF [138, 139].

## CHALLENGES IN PREVENTING AND TREATING EVD

Vaccines for humans are currently available for a minority of the hemorrhagic fever viruses, including Hantaan virus, DENV, tick-borne encephalitis virus, YFV, and EBOV. Currently 2 recombinant vaccines against EBOV are produced: the single-dose Ervebo (rVSVΔG-ZEBOV-GP), approved by the US Food and Drug Administration, and the heterologous prime boost Zabdeno/Mvabea (Ad26.ZEBOV and MVA-BN-Filo), approved by the European Medicines Agency [140]. Other recombinant vaccines against EBOV are available in China and Russia; yet, relatively little is known about them, and they are not generally available outside of their manufacturing countries. At present, only Ervebo is backed by any published clinical efficacy data. The first such study was a cluster-randomized ring vaccination trial conducted in Guinea and Sierra Leone at the end of the 2013–2016 West Africa EVD epidemic [141]. It reported a perfect vaccine efficacy of 100%, although the ring vaccination design raised concerns related to the accuracy of this result, particularly due to differences in how the clusters received starkly different follow-up care that may have affected further EBOV transmission and led to an overestimated vaccine efficacy [142]. During the 2018–2020 EVD outbreak in the DRC, the PALM clinical trial—which was conducted to evaluate various monoclonal antibodies and the antiviral drug remdesivir as potential therapeutics for EVD—noted that numerous patients with EVD had indeed been vaccinated with Ervebo, with many of these reporting vaccination >10 days prior to enrollment [143]. This prompted further retrospective analysis into the vaccine's efficacy, resulting in 2 additional studies published in 2024: the first was a retrospective cohort analysis that reported that patients with EVD who had received Ervebo had improved survival and lower viremia; the second was a retrospective test-negative study that estimated the overall vaccine efficacy of Ervebo at 84% at ≥10 days following administration [144, 145]. The unusual and suboptimal design of these studies in comparison with a gold standard randomized, placebo-controlled, double-blind efficacy trial underscores the great difficulty in evaluating EVD vaccines: the unpredictability and relative infrequency of EVD outbreaks effectively make undertaking such studies impossible. While the mere existence of vaccines for EVD is immensely encouraging and testament to the investments made over the past decade, great care must be taken, given our lack of satisfactory data, to ensure that a false sense of confidence is not conveyed regarding the vaccine's efficacy or durability. Any suggestion that could lower the threshold of precaution taken during EVD outbreaks must be avoided [142]. Although future circumstances may provide opportunities for improved data gathering, important questions regarding vaccination for EVD are likely to remain unanswered for the time being.

The small molecule antiviral drug remdesivir had demonstrated promising preclinical data in nonhuman primates for treating EVD, although human data are limited and a clear benefit is yet to be shown [146]. However, development and testing of monoclonal antibodies for the treatment of EVD have been more promising over the past decade. The PALM clinical trial in the DRC showed the most benefit from treatment with either the single monoclonal antibody mAb114 or the monoclonal antibody cocktail REGN-EB3, with overall case fatality rates of 35.1% and 33.5% among the treatment groups, respectively. Outcomes for these two groups were more favorable than the groups treated either with ZMapp (another monoclonal antibody cocktail) or with remdesivir, which had overall case fatality rates of 49.7% and 53.1%, respectively [143]. Although these data are encouraging, the overall case fatality rates in both the mAb114 and REGN-EB3 treatment groups still exceed those of most other VHFs, even in the absence of targeted therapeutics. The odds of death increased by 11% for each day that treatment was delayed, and patients with an initial diagnostic cycle threshold value ≤22 were 4 times more likely to die. Furthermore, even when treated with mAb114 or REGN-EB3, patients who presented later in the course of illness, as is often the case in EVD outbreaks, or with higher viral loads had a combined case fatality rate of 67%, demonstrating little, if any, benefit of these therapies in fulminant EVD [143]. Adding uncertainty to this overall picture, a separate trial assessing ZMapp that was conducted in West Africa showed a case fatality rate of 22% in the treatment group as compared with the control group at 37%, which received current standard of care [147]. The reasons for the vastly discrepant results between trials are not clear.

## CHALLENGES IN RESEARCH AND HEALTH CARE INFRASTRUCTURE FOR EVD

While the goal of vaccination against EVD is to reduce overall case numbers and disease severity and targeted therapeutics are possibly proving useful for treatment, the mainstays of managing EVD outbreaks remain coordinated public health efforts to reduce transmission and supportive care measure to treat patients. Both mainstays depend on robust infrastructure and highly trained personnel. EVD outbreaks often occur in some of the most remote resource-limited areas in the world. The minimal access to health care facilities, limited communication and transportation services, and complex social and economic factors that are present in these locations can greatly hinder public health efforts to isolate the sick and effectively deliver lifesaving messages to the general population. Many of the complications that can arise in EVD, such as respiratory failure or renal failure, require technologically advanced and invasive supportive care measures [148, 149]. In some instances, simply establishing intravenous access for patients with EVD during outbreaks was not feasible, underscoring the somewhat daunting hurdles to utilize measures such as ventilatory support or dialysis. Adequate testing and surveillance are also challenging, and delays in detection of EVD may lengthen and ultimately enlarge outbreaks [106].

EBOV is considered a tier 1 select agent by the US Department of Health and Human Services and a category A biological agent by the US Centers for Disease Control and Prevention [150]. As such, work involving EBOV (and all other viruses within the family Filoviridae) must be conducted exclusively at the highest level of containment utilizing biosafety level 4 (BSL-4) facilities and procedures. This contrasts with the majority of the other hemorrhagic fever viruses, aside from a handful of viruses that also require BSL-4 containment within the families Arenaviridae (eg, LASV, Junín virus) and Flaviridae (eg, Kyasanur Forest disease virus, Alkhumra hemorrhagic fever virus), for which work can safely be conducted at BSL-3 or BSL-2 [150]. Only a few dozen BSL-4 facilities are currently in existence, the majority of which are in Europe and the United States, far from the known geographic range of EBOV [151]. Thus, much of the basic and translational research involving EBOV is a niche endeavor that is largely inaccessible for most scientists. While construction of new BSL-4 facilities in central or West Africa may currently be unnecessary or impractical for numerous reasons, investment in continued improvement of the research and health care infrastructure in areas most affected by EVD outbreaks will empower local scientists and HCWs to be better positioned to provide critical laboratory investigations and advanced patient care within their own localities.

## CONCLUSION

VHF is caused by numerous emerging and reemerging zoonotic viruses across 6 viral families. Among these, EBOV poses a unique set of challenges that demand ongoing, multifaceted, and multinational investment. Despite being discovered nearly 50 years ago, very little is understood regarding the ecology of EBOV and the circumstances and drivers of spillover to humans. Definitive identification of the natural reservoirs of EBOV and characterization of its enzootic cycles and spillover dynamics are critical components to preventing future outbreaks and epidemics. EBOV's ability to readily transmit directly from human to human following spillover, combined with the extraordinarily high case fatality rates of infection, makes containment efforts extremely difficult and poses significant risks to HCWs. Developments in vaccines and targeted therapeutics for EVD are monumental accomplishments to help stem the transmission of EBOV and provide improved outcomes for patients with EVD, but they are hindered by the challenges of conducting robust studies that can more adequately assess the efficacy of these medical countermeasures to provide unambiguous guidance on best practice. Perennial hurdles in infrastructure must also be overcome by expanding the health care and research capacity in areas most affected by EVD to allow for improved public health and clinical responses to outbreaks and to facilitate advanced research capabilities. Confronting these challenges cannot wait until the next EVD epidemic.
